# Supplementary material for: Alterations in Anandamide Synthesis and Degradation during Osteoarthritis Progression in an Animal Model
Source: Int J Mol Sci. 2020 Oct 6;21(19):7381. doi: 10.3390/ijms21197381 (PMC7582975; doi:10.3390/ijms21197381)
Supplement: Supplementary file 1 [file ijms-21-07381-s001.pdf]

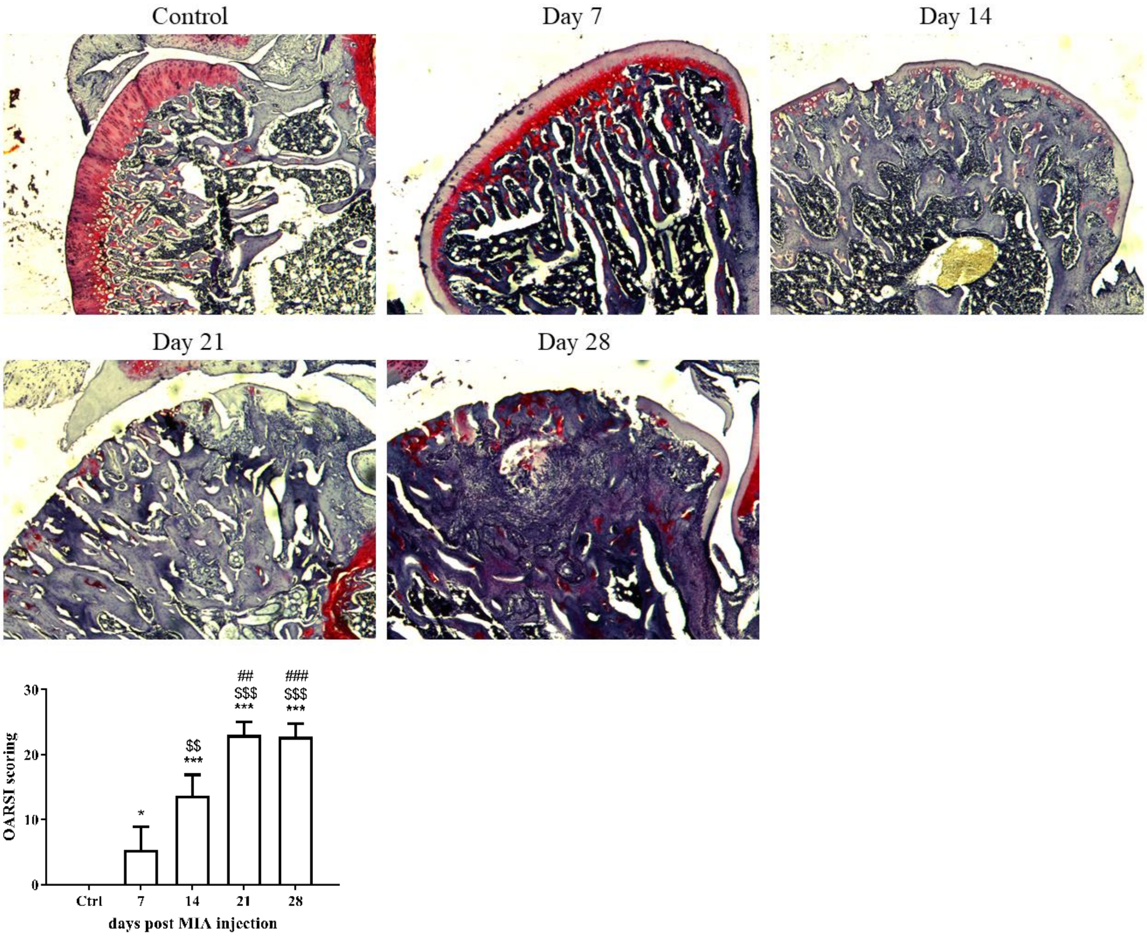


**Supplementary figure 1.** Histological evaluation of rat's knee cartilage following osteoarthritis induction. The samples were collected 7, 14, 21 and 28 days after 1 mg MIA injection; the control group did not receive any treatment. Bar chart representation of OARSI scoring from abovementioned groups results are presented as means ± SEM from a group of 3–6 samples. Statistical analysis was performed using one-way ANOVA followed by Dunnett post hoc-test (intact animals treated as a control group). * denotes $p < 0.05$; ** denotes $p < 0.01$; *** denotes $p < 0.001$ vs. intact animals; $ vs. day 7; # vs. day 14.

Histological analysis—methods

Histological analysis was performed on the sagittal sections of the femoral condyles. Samples have been decalcified in 10% formic acid for 10 days, processed through increasing concentrations of saccharose, embedded in Leica OTC tissue freezing medium and frozen in liquid nitrogen. A Leica cryostat was used to make 10 µm coronal sections through the entire cartilage at 45 µm intervals yielding 10–13 different levels. Sections were then stained with Safranin O and hematoxylin according to the standard techniques. Histological evaluation of the severity of osteoarthritis was performed by an observer, who was blinded to the pharmacological treatment, according to the Osteoarthritis Research Society International (OARSI) scoring system [1].

1 K. P. H. Pritzker et al., "Osteoarthritis cartilage histopathology: Grading and staging," Osteoarthr. Cartil., 2006, doi: 10.1016/j.joca.2005.07.014.
